# Relaxation Processes of Food Hydrocolloids in Diluted, Semidilute, and Concentrated Solutions: NMR and Hydrodynamic Approach

**DOI:** 10.3390/polym17243326

**Published:** 2025-12-17

**Authors:** Magdalena Witek, Anna Ptaszek, Paweł Ptaszek

**Affiliations:** 1 Department of Biotechnology and General Technology of Food, Faculty of Food Technology, University of Agriculture in Krakow, ul. Balicka 122, 30-149 Krakow, Poland; magdalena.witek@urk.edu.pl; 2 Centre of Innovation and Research on Prohealthy and Safe Food, University of Agriculture in Krakow, ul. Balicka 104, 30-149 Krakow, Poland; 3 Department of Engineering and Machinery in Food Industry, Faculty of Food Technology, University of Agriculture in Krakow, ul. Balicka 122, 30-149 Krakow, Poland

**Keywords:** relaxation, dynamic light scattering (DLS), nuclear magnetic resonance (NMR), hydrodynamic properties, guar gum, xanthan gum

## Abstract

The paper presents the results of relaxation studies for aqueous solutions of guar gum (GG) and xanthan gum (XG) in diluted, semidilute, and concentrated ranges over a wide temperature range (20–90 °C). Relaxation studies were performed using NMR and DLS methods. Due to variations in the biopolymer–biopolymer interactions, XG chains formed a more complex structure in solution than GG chains did. Consequently, differences in $T_{2}$ relaxation times were observed in the diluted and semidilute regions. Comparing the autocorrelation functions of XG and GG solutions in the semidilute region revealed differences in their relaxation behaviour.

## 1. Introduction

Relaxation is a group of phenomena that are related to the interactions between molecules (structures that they create) when these have been subjected to energy input (mechanical, thermal, etc.) [1,2]. It describes the behaviour of system after this trigger has disappeared. Knowledge of the qualitative and quantitative aspects of relaxation phenomena is important not only from a scientific point of view, but also in industrial practice. It provides insight into the structure of a food product and the quality of interactions between its components, especially between structure-forming additives and water. This enables the shaping of product properties and the selection of production (technological) processes and storage conditions [3,4]. The most important external factors that strongly influence the interactions between food product components are temperature (thermal processes and storage conditions) and shearing/stress (transport processes, mixing, dosing, etc.). In the case of mechanical processes, relaxation can be studied using rheological methods, and information on material constants (e.g., relaxation time and retardation time) can be obtained [5,6,7]. These values are determined using phenomenological models that describe the behaviour of the product structure. Although this provides a macroscopic view, it allows one to track the influence of temperature and shear conditions on the properties of the food product. In terms of food production, the main focus is on studying the effect of structuring additives on the mechanical properties of food products, which helps to determine its optimal concentration to ensure the desired mechanical and textural properties of the final product [4]. At a microscopic level, the properties of a product are determined by the interactions between its components, particularly the interactions between structure-forming additives (mainly polysaccharides) and water [8,9,10,11]. These interactions largely determine the product’s physical stability during shearing, stress, or temperature changes [3,12]. Furthermore, once these two factors have disappeared, it is important to understand the timescale over which undesirable phenomena such as phase separation or syneresis may occur [13]. The role of polysaccharides as structure-forming and water-binding components has been widely documented in the scientific literature [14,15,16]. Their selection is mainly based on the structure of the chain formed by monosaccharides (e.g., glucose, mannose, or galactose), which are linked by glycosidic bonds. Type 1–4 bonds form the linear part of the chain, while type 1–6 bonds form side branches with different molecular characteristics containing functional groups (e.g., hydroxyl, amide, carboxyl, or acetyl). These functional groups give polysaccharides their unique properties in aqueous solutions, including those characteristic of polyelectrolytes, and determine the nature of polymer–solvent interactions. The second factor is the concentration of the biopolymer, which, together with its affinity for the solvent, determines polymer–polymer interactions. Due to the nature of the interactions involved in the physical chemistry of polymers, three concentration ranges can be distinguished: dilute, semidilute, and concentrated. These ranges are separated by two critical concentrations. Given the need for a more comprehensive understanding of relaxation phenomena in water-containing polysaccharide systems, NMR methods provide important analytical capabilities. NMR methods are used to determine the chemical structure of polysaccharides and to detect them in food products, but there has also been growing interest in using them to study interactions in polysaccharide–water systems in recent years [17,18,19,20,21]. The most popular polysaccharides in food industry belong to polyselectrolite xanthan gum (XG) and nonionic guar gum (GG). Xanthan gum can exist in various conformations: native (helical), denatured (coil), and renatured [22]. The third conformation: renaturated is obtained by heating and cooling xanthan system [22]. Due to the complex nature of xanthan gum–water interactions depending on temperature, numerous publications refer to the first and second critical concentration of XG solutions [23,24,25]. Unlike ionic xanthan, guar gum macromolecules adopt an elastic coil conformation in water solutions, form a cross-linking network, and create high-viscosity solutions [26,27,28]. The properties of guar gum (GG) and xanthan gum (XG) have been studied by high-resolution high-field spectroscopy of NMR of different nuclei to look for structural changes during different modifications in dilute and semi-dilute systems [29,30,31,32,33,34,35]. Apart from molecular structure of xanthan and guar gums derived from high resolution spectra, the dynamics of the system can be studied by measurements of proton NMR spin–spin ($T_{2}$) and spin–lattice ($T_{1}$) relaxation times [36,37]. The relaxation times measured in the low field NMR are mainly originated from water protons that interact with protons of gums in solutions. There are few studies concerning applications of low field NMR relaxation studies in xanthan and guar gum solutions [38,39]. In study [39], the NMR spin–spin relaxation time and rheology study showed that guar gum behaved as a typical solution regardless temperature in comparison to scleroglucan of same concentration that formed gel.

Relaxation phenomena in xanthan and guar gum solutions have not been extensively investigated using low-field nuclear magnetic resonance (NMR) [38,39]. There are also no reports in the literature on how temperature affects the relaxation of these polysaccharide biopolymer chains. Additionally, the relaxation phenomenon is not mentioned in relation to rheological properties, including hydrodynamic properties, in the dilute and semidilute regions, as determined by dynamic light scattering (DLS) and rotational rheology.

This study aimed to investigate the relaxation phenomena of selected biopolymer chains in aqueous solutions using NMR and DLS techniques. Measurements were taken for two popular food additives, xanthan and guar gum. The NMR studies were carried out for all selected regimes while DLS for diluted and semidilute solutions.

## 2. Materials and Methods

### 2.1. Hydrocolloids

Commercially available, food-grade powdered gum preparations: guar (GG) and xanthan (XG) (Hortimex Sp. z o.o., Konin, Poland) were used in this study. Chromatographic analyses of hydrocolloids were performed by gel permeation chromathography (GPC) method according to the procedure [40]. The average weight molecular masses were 2000 kDa for xanthan gum and 750 kDa for guar gum, respectively.

### 2.2. Preparation of the Solutions

Powdered preparations were used to prepare aqueous solutions at concentrations of 0.5, 0.05, and 0.01 g/dL for xanthan gum and 2.5, 0.5, and 0.01 g/100 mL for guar gum. In order to rehydrate the gum preparations, the treatment of the solutions involved two steps. In the first stage, the gums were mixed using rotational stirrer (500 rpm) for 0.5 h at ambient temperature. In the second stage, the obtained solutions were mixed and heated at the temperature of 45 °C for 2 h. The range of solution concentrations was selected based on the literature data concerning the relationship between average weight molecular mass and critical concentrations. For XG, the critical concentrations took the values of 0.023 g/dL for c* and 0.07 g/dL for c**, respectively [24]. In the case of guar gum, c* = 0.28 g/dL [27].

### 2.3. Nuclear Magnetic Resonance (NMR)

Proton relaxation measurements were performed using a Bruker Minispec NMR spectrometer (Bruker BioSpin, Poznan, Poland) operating at the resonance frequency of 60 MHz at stabilized temperatures in the range from 20 °C to 90 °C with a heating/cooling rate of 2.5 K/min. Carr–Purcell–Meiboom–Gill (CPMG) spin-echo sequence was used to obtain T2 with a delay time between the 90° and 180° pulses equal to 2 ms and 4096 echoes used. Data were averaged over 4 scans with a recycle delay time of 15 s. $T_{2}$ was measured for three xanthan gum (0.5, 0.05, and 0.01 g/dL) and two guar gum (2.5 and 0.5 g/100 mL) aqueous solutions. The $T_{2}$ decays were fitted by single exponential function, as given by the equation:

(1) $$F\left(t\right)=L\cdot e^{-\frac{t}{T_{2}}}$$

where *L* is the amplitude and $T_{2}$ is the spin–spin relaxation time.

Dipole–dipole relaxation is dominant in water and in biopolymer solutions. For identical spin systems interacting via dipolar couplings at fixed intermolecular distances r and undergoing reorientation with a characteristic correlation time $\tau_{c}$, the spin–spin relaxation rate ($\frac{1}{T_{2}}$), measured at the proton resonance frequency $\omega_{0}$, is described by [41]:

(2) $$\frac{1}{T_{2}}=\frac{A}{2}\cdot \left(3\tau_{c}+\frac{5\tau_{c}}{1+\omega_{0}^{2}\tau_{c}^{2}}+\frac{2\tau_{c}}{1+4\omega_{0}^{2}\tau_{c}^{2}}\right)$$

where $A=\frac{3\gamma^{4}\hbar^{4}}{10r^{6}}{\left(\frac{\mu_{0}}{4\pi }\right)}^{2}$, and $\mu_{o}$ and γ denote the magnetic permeability of vacuum (magnetic constant), and gyromagnetic ratio of nucleus, respectively, while $\hbar =\frac{h}{2\pi }$, *h* is the Planck constant. For fast molecular motions, where $\omega_{0}^{2}\tau_{c}^{2}\ll 1$, $\frac{1}{T_{2}}=5A\tau_{c}$ and then the relaxation rate is proportional to correlation time $\tau_{c}$. The relaxation time depends, in turn, on the temperature through the Arrhenius equation:

(3) $$\tau_{c}=\tau_{c0}e^{\frac{E_{a}}{RT}}$$

where $E_{a}$ is the activation energy associated with molecular motions reflected in $T_{2}$ relaxation and R is the universal gas constant.

In this work, an Arrhenius-type dependence over the entire temperature range was used to fit log($T_{2}$)–reciprocal temperature dependence, which can be expressed as

(4) $$log\left(T_{2}\right)=log\left(T_{20}\right)-\frac{E_{a}}{RT}$$

where log($T_{20}$) is the logarithm of the pre-exponential factor, containing constant *A* and $\tau_{c0}$.

### 2.4. Dynamic Light Scattering (DLS)

The biopolymer solutions were tested using the Backscattering Mode technique at stabilized temperatures in the range from 20 °C to 90 °C with a heating/cooling rate of 2.5 K/min, on an Diffusing Wave Spectroscopy (DWS) RheoLab (LS Instruments AG, Fribourg, Switzerland) with the help of two-cell-echo technology for non-ergodic samples. The DWS equipment with the option of Backscattering Mode was used because chains larger than 200 nm were expected in the tested biopolymer solutions. This measurement method enabled colloidal solutions of up to 1000 nm to be measured. As a source of light, a solid-state laser with output power of 45 mW at λ = 685 nm was used. The light scattering angle θ for measurements was 173°. Three repetitions were performed for all samples. The time average vertical intensity correlation function $g^{2}\left(\tau \right)-1$ was obtained with the acquisition time of 120 s for each run with the help of LsLab Software. There were two mathematical models tested [42]. The first was the Kohlrausch–Williams–Watts (KWW) stretched exponential function:

(5) $$g^{2}\left(\tau \right)-1\approx {\left\{a\cdot exp\left(\frac{-\tau }{\tau_{f}}\right)+\left(1-a\right)\cdot exp{\left[-\left(\frac{\tau }{\tau_{s}}\right)\right]}^{\beta }\right\}}^{2}$$

where $g^{2}\left(\tau \right)-1$ is the intensity autocorrelation function, $\tau_{f}$, $\tau_{s}$ are relaxation times of the fast and slow components, respectively, β is the exponent of the stretched exponential, τ is the delay time, and *a* and (1−a) represent the fractional contribution of the two processes. The estimation of parameters (Equation (5)) was according to the Levenberg–Marquardt algorithm using the least squares method. The diffusion coefficients for slow $D_{s}$ and fast $D_{f}$ components were calculated according to equations:

(6) $$D_{k}=\frac{1}{\tau_{k}\cdot q^{2}},k=s,f$$

where $q=\frac{4\pi n}{\lambda }\cdot sin\left(\frac{\theta }{2}\right)$ is s the value of magnitude of the scattering wave vector. Second one was the exponential model with the parameter describing the fractal dimension of scattered photons:

(7) $$g^{2}\left(\tau \right)-1\approx {\left\{a\cdot exp\left(-D_{A}q^{2}\tau \right)+\left(1-a\right)\cdot exp{\left[1+\left(\frac{\tau }{\tau *}\right)\right]}^{\frac{n-1}{2}}\right\}}^{2}$$

where: τ* is the characteristic time where the power law behavior appeared and *n* is the fractal dimension of scattered photons n∈(0,1), τ is the delay time, and *a* and (1−a) represent the fractional contribution of the two processes (*slow* and *fast*). Estimation of parameters (Equation (5)) was conducted according to the Levenberg–Marquardt algorithm using the least squares method:

(8) $$\chi^{2}=\Sigma {\left\{\left[g^{2}\left(\tau \right)-1\right]-{\left[g^{2}\left(\tau \right)-1\right]}_{experim}\right\}}^{2}⟶min$$

The values of hydrodynamic radius $R_{h}$ were calculated according to Stokes–Einstein equation:

(9) $$R_{h}=\frac{k_{B}\cdot T}{6\pi \eta_{sol}\cdot D}$$

where $k_{B}$ is the Boltzmann constant, *T* is the absolute temperature on the Kelvin scale, and $\eta_{sol}$ is the water viscosity at temperature *T*. *D* is the value of diffusion coefficient obtained from the Equation (6) or direct from Equation (7).

### 2.5. Data Analysis

The method of data analysis was based on the Marquardt–Levenberg nonlinear least-square iteration procedure. Calculations were carried out using software prepared in Python 3.11.4.

## 3. Results

### 3.1. NMR Relaxation

**Figure 1:**
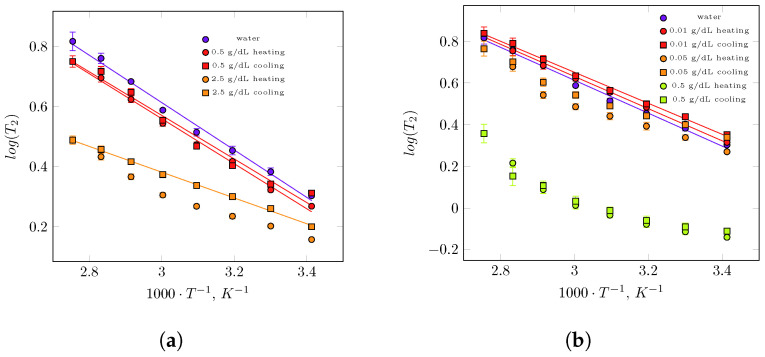
Spin–spin relaxation time $T_{2}$ vs. reciprocal temperature for guar gum solution of concentration 0.05 g/dL—semidiluted regime and 2.5 g/dL—concentrated regime (**a**) and xanthan gum solutions in three regimes: diluted—0.01 g/dL, semidilute—0.05 g/dL and concentrated—0.5 g/dL (**b**). Additionally, on both figures, the temperature dependence of $T_{2}$ for water has been shown. Solid lines are Arrhenius fits of data in the whole temperature range.

Time-domain measurements showed a single relaxation time $T_{2}$ of water protons for all concentrations of guar and xanthan gums (Figure 1). The single-exponential character of the $T_{2}$ decay, in contrast to what has been reported for some polysaccharide–water systems [43,44,45,46], suggests that, on average and over the experimental timescale, all water molecules in guar and xanthan gum solutions exhibit similar dynamics. Any distinct water populations that may exist in these hydrophilic polymer solutions must therefore exchange faster than the timescale of the magnetization decay measurement. This interpretation is also consistent with the observation of single relaxation times reported for certain other polysaccharide systems [47,48,49]. As expected, $T_{2}$ increases with an increase in temperature, indicating the overall greater mobility of water. The relaxation time of the protons of the gums in solution is very short and is not detected in CPMG experiment, but the influence of these is noticeable in the observed values $T_{2}$. Their values decreased (i.e., relaxation was faster) as the gum concentration increased. The spin–spin relaxation time decreased from 1855 ms for concentration 0.5 g/dL to 1436 ms for 2.5 g/dL at 20 °C. The greater the concentration of gum in the water, the larger the reduction in relaxation time. A similar trend in the reduction in relaxation time has also been observed for xanthan gum solutions. When moving from the dilute through the semidilute to the concentrated regime, the $T_{2}$ values decrease from 2068 ms to 1864 ms and further to 756 ms at 20 °C. It should also be noted that at this temperature, the $T_{2}$ value of xanthan gum is approximately 2.5 times lower than that of guar gum at the same concentration of 0.5 g/dL.

Figure 1 shows the $T_{2}$ times across the temperature range between 20 °C and 90 °C that are presented in a standard plot of $\log T_{2}$ on a reciprocal of temperature in K for all studied solutions of guar and and xanthan gums in heating and cooling regimes. Additionally, the temperature dependence of pure water is presented in Figure 1, and $T_{2}$ generally decreases as the reciprocal of temperature increases, which indicates reduced mobility of water molecules. For most gum solutions studied, $T_{2}$ follows an Arrhenius-type dependence over the entire temperature range. From the logarithmic form of this equation, activation energies were obtained. For a low concentration of guar gum in water, the spin–spin relaxation time increases approximately threefold as temperature rises from 20 °C to 90 °C. The process is reversible during cooling, a decrease in molecular mobility of water protons was observed, and the temperature dependence of $T_{2}$ follows almost the same course as during in heating. For the 0.5 g/dL guar gum solution, the $T_{2}$–temperature curve in both heating and cooling regimes lies slightly below that of pure water. This relaxation behaviour suggests that this system behaves as an aqueous polymer solution with relatively weak water–polymer interactions. The activation energy extracted from Arrhenius fits showed only minor difference between pure water (15.1 kJ/mol) and the 0.5 g/dL guar gum solution in heating (14.4 kJ/mol) and cooling (13.9 kJ/mol). Stronger water–polymer interactions are expected at higher guar gum concentrations (2.5 g/dL). Across the whole temperature range, $T_{2}$ is shorter for the more concentrated solution, indicating higher viscosity compared with the 0.5 g/dL system. $T_{2}$ increases approximately twofold between 20 °C and 90 °C, and the activation energy decreases to 9.4 kJ/mol for heating and 8.2 kJ/mol for cooling. The lower $E_{a}$ values correspond to water molecules diffusing within a denser polymer network characterized by slower polymer dynamics. It should also be noted that a significant difference in $T_{2}$ appears during the cooling cycle. For a given temperature, relaxation times are longer upon cooling, which indicates higher water mobility due to weaker water–polymer interactions during this stage.

The spin–spin relaxation time as a function of temperature for three xanthan gum solutions have been shown in Figure 1b. For the 0.5 g/dL guar gum, the spin–spin relaxation time $T_{2}$ increased approximately threefold as the temperature rose from 20 °C to 90 °C, which can be explained by the increase in molecular motion (i.e., the shortening of correlation times). This process is reversible: during cooling, a decrease in molecular mobility of water protons was observed, and the temperature dependence of $T_{2}$ followed almost the same trend as during heating. The $T_{2}$–temperature curve in both heating and cooling regimes lies slightly below that of pure water. This relaxation behaviour suggests that this system perform as an aqueous polymer solution with relatively weak water–polymer interactions. The activation energies extracted from the Arrhenius fits show only minor differences between pure water (15.1 kJ/mol) and the guar gum solution during heating (14.4 kJ/mol) and cooling (13.9 kJ/mol). Stronger water–polymer interactions are expected at higher guar gum concentrations (2.5 g/dL). Across the entire temperature range, $T_{2}$ was shorter for this solution, indicating higher viscosity compared with the 0.5 g/100 mL system. In the heating cycle, $T_{2}$ increased approximately twofold between 20 °C and 90 °C, but this increase deviated from linearity. A significant difference in $T_{2}$ appeared during the cooling cycle: for a given temperature, relaxation times were longer upon cooling, indicating higher water mobility due to weaker water–polymer interactions during this stage. Moreover, the $T_{2}$–reciprocal temperature dependence was Arrhenius-type, for which the activation energy was calculated as 8.2 kJ/mol. The lower $E_{a}$ values correspond to water molecules moving within a denser polymer network characterized by slower polymer dynamics. For the most diluted system, characterized by a 0.01 g/dL xanthan gum concentration, the temperature dependence of $T_{2}$ followed the Arrhenius behaviour observed for pure water Figure 1, with similar activation energies of 14.8 kJ/mol during heating and 14.2 kJ/mol during cooling cycles. This suggests that, in this case, water does not sense the influence of the polymer. In the semidiluted system, the dependence of relaxation time on reciprocal temperature began to deviate from Arrhenius-type plot, both in heating and cooling regimes. The relaxation times decreased only slightly compared with the diluted system, but the for 0.05 g/dL xanthan gum solution, a clear hysteresis can be observed, resembling the $T_{2}$–reciprocal temperature behaviour of the 2.5 g/dL guar gum system. However, the main difference between these two biopolymer solutions lies in the absolute values of relaxation times: a higher water mobility in xanthan gum solutions was evident. For the most concentrated xanthan gum solution (0.5 g/dL), $T_{2}$ values still increased with temperature; however, the deviation from an Arrhenius behaviour becomes even more pronounced. Interestingly, no hysteresis was observed in the heating–cooling cycle for this concentration, indicating that water dynamics do not change during the thermal cycle. The non-Arrhenius behaviour, however, suggests that additional factors govern water mobility in this highly concentrated xanthan gum system.

### 3.2. Hydrodynamic Properties of the Solutions

**Figure 2:**
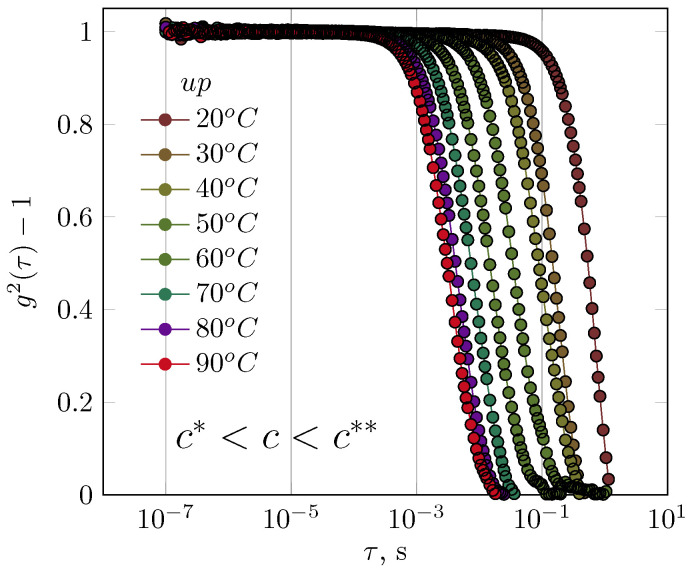
The influence of temperature on the course of the autocorrelation functions for guar gum solution (concentration of 0.5 g/dL—semidilute regime).

First, the results for GG solutions in the semidilute range are presented. It was not possible to obtain interpretable results of DLS measurements for concentrated GG solutions. An increase in temperature caused changes to the autocorrelation function (Figure 2). At the lowest temperature, this function disappeared after 1 s; at 90 °C, however, the relaxation of the GG chains occurred within a shorter time interval of 0.01 s. Changes in the behaviour of GG chains in the semidilute regime occurred evenly as a function of temperature without altering the shape of the autocorrelation function (Figure 2).

The autocorrelation function was not presented for the experiment in which the temperature was reduced from 90 °C to 20 °C. Although clear hysteresis was observed in the autocorrelation function, the authors concluded that this was due to the inertia of cooling of the sample and not to changes in the relaxation of the solution structure; despite the relatively low concentration of GG, repeatability of the autocorrelation function could not be obtained.

**Figure 3:**
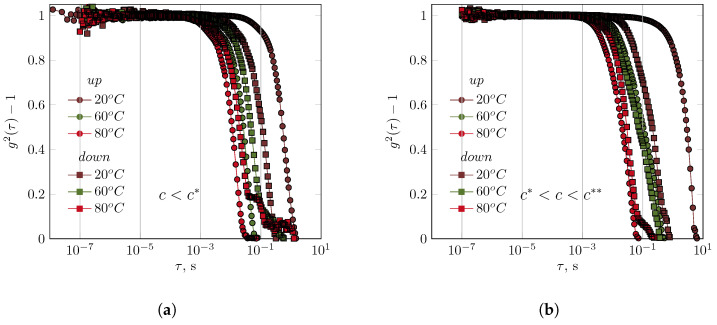
The influence of temperature on the course of the autocorrelation functions for xanthan gum solutions (concentrations of (**a**) diluted regime: 0.01 g/dL, and (**b**) semidilute regime: 0.05 g/dL).

The XG solution (0.01 g/dL) exhibited behaviour similar to that of the GG solution, even though it was in the diluted range (Figure 3).

This solution had a concentration 50 times lower than that of the GG solution. The nature of the autocorrelation function remained unaffected by temperature. Comparing the autocorrelation functions at the stages of the experiment with increasing and decreasing temperatures revealed different behaviors in the solutions. After being cooled to 20 °C, the relaxation of the XG chains in a 0.01 g/dL solution occurred faster than in the initial phase of the experiment. However, at 60 °C and 80 °C, the opposite behavior was observed (Figure 3). At 60 °C, the relaxation phenomena during the cooling of the solution occurred according to a complex mechanism, as evidenced by the autocorrelation function. Increasing the XG concentration to 0.05 g/dL (semidilute) slightly shortened the relaxation times as a function of temperature. It was not possible to obtain interpretable results of DLS measurements for concentrated XG solutions.

Two functions were fitted to the measurement data: the KWW equation (Equation (5)) and the exponential function (Equation (7)). In the case of the KWW function, relaxation times for the fast and slow modes were estimated; however, unfortunately, these were physically uninterpretable. The values of hydrodynamic radii ($R_{h}$—Equation (9)) calculated from relaxation times were extremely high, more than 1000 nm. This indicated that the solution contained a phase exhibiting behaviour characteristic of gel-like clusters. Attempting to apply the second equation was successful. This equation is applicable to systems in which a gel or pseudo-gel phase is present. This means that diffusion coefficients with extremely small values can be estimated. In the case of the GG solution, the $R_{h}$ values were greater than 1000 nm only at a temperature of 20 °C. As the measurement temperature increased, these values decreased from 570 nm at 30 °C to 11 nm at 90 °C (Table 1). The characteristic time (τ*) values were greater than 1000 s due to the shape of the autocorrelation function, which is characteristic of gelling systems.

Analysis of changes in $R_{h}$ values (Table 1) indicates a phenomenological similarity in the behaviour of GG (0.5 g/dL) and XG (0.01 g/dL) solutions. In both cases, as *T* increased, the $R_{h}$ values decreased significantly within the same numerical range (from 350 nm at 40 °C to approximately 20 nm at 90 °C). Changing the concentration of the solution from diluted (0.01 g/dL) to semidilute (0.05 g/dL) resulted in a fundamental change in the behaviour of XG solutions. Within the temperature range of 20–50 °C, the Rh values were several times greater at 1000 nm. Near the helix–coil transition temperature (50–60 °C), there was a sharp decrease in $R_{h}$ values.

## 4. Discussion

When analysing the NMR relaxation data at 20 °C, it can be concluded that both guar gum and xanthan gum show a concentration-dependent effect on relaxation times, although the nature of this correlation differs between the two systems. For the same concentration (0.5 g/100 mL), the relaxation times observed for xanthan gum were significantly shorter than those for guar gum. The shorter relaxation times in xanthan gum solutions may be attributed to its higher molecular weight and its more complex, helical conformation. In xanthan gum systems, proton exchange between polymer protons and water protons appears to play a substantial role in accelerating the transverse relaxation of water. These observations are consistent with the physical properties of xanthan gum, which is known to exhibit high viscosity even at relatively low concentrations [50]. An increase in temperature altered the behaviour of the GG chains, differentiating them according to concentration. The difference in the temperature dependence between heating and cooling indicates the presence of thermal hysteresis. The longer $T_{2}$ values observed during cooling 2.5 g/dL solution suggest that the polysaccharide network does not fully resemble upon temperature decrease, resulting in increased water mobility and reduced dipolar interactions compared to the heating cycle. The Arrhenius-type temperature dependence with reduced activation energy of $T_{2}$ indicates that water dynamics in guar gum matrix become less temperature-dependent, reflecting a more structurally stable microenvironment in cooling cycles. The incomplete recovery of water–polymer interactions was also observed for xanthan gum, although in the semidiluted concentration (0.05 g/dL) range and without a Arrhenius-type behaviour in either heating or cooling regimes. The relatively long relaxation times measured for this solution reflect a less constrained microenvironment, with more thermally responsive biopolymer–water interactions. In other words, in a less rigid polysaccharide network, biopolymer–water interactions can be easily disrupted or reorganized upon heating. Xanthan gum exhibits markedly different relaxation behavior in the concentrated regimes (0.5 g/dL), where the deviation from Arrhenius temperature dependence of $T_{2}$ was most apparent. This suggests that the dynamics of water and polymer become more complex and, as in any non-Arrhenius type behaviour, cannot be described by a single activation energy. Despite the complex kinetics, the system returns to the same state after heating and cooling (absence of hysteresis), indicating that the polymer structure and water mobility restrictions are reversible and reproducible. In this regimes, $T_{2}$ reflects the combined contributions of water populations with different correlation times, resulting in a nonlinear (non-Arrhenius) temperature dependence.

Differences in interactions with water were evident in the osmotic pressure measurement and for data calculated from the values of the second virial coefficient ($A_{2}$) for diluted GG and XG solutions [51]. While the values of the second virial coefficient were negative in both cases (indicating stronger biopolymer–biopolymer interactions than biopolymer–water interactions), the $A_{2}$ value was small and negative for diluted GG solutions, in contrast to XG solutions for which the $A_{2}$ value was negative but two orders of magnitude greater in absolute value. XG solutions were found to have a greater ability to absorb water, as evidenced by their higher reduced osmotic pressure values compared to GG solutions. Due to variations in the interactions between biopolymers–water [51], XG chains formed a more complex structure in solution than GG chains did. Consequently, differences in $T_{2}$ relaxation times as a function of reciprocal temperature were observed in the diluted and semidilute, as well as in concentrated regions (Figure 1). In general, increasing the XG concentration causes a systematic transition of $T_{2}$(T) from an Arrhenius profile without hysteresis (dilute regime), through an non-Arrhenius profile with hysteresis (semidilute regime), to a non-Arrhenius profile without hysteresis (concentrated regime). Comparing the autocorrelation functions of XG and GG solutions in the semidilute region revealed differences in their relaxation behaviour. Changes in temperature affected GG solutions to a greater extent than XG solutions. GG solution autocorrelation functions disappear over a wider range of relaxation times (Figure 2) than XG solution autocorrelation functions (Figure 3b) under the same overlapping conditions. Similar behaviour can be obtained in terms of biopolymer–biopolymer interactions and resulting interaction decay times (the course of autocorrelation functions) using XG in the dilute regime and GG in the semidilute regime. Studies using measurements in the area of linear viscoelasticity [40] and flow [52,53,54] revealed differences in the relaxation of GG and XG solutions at concentrations corresponding to the concentrated range. In the case of concentrated GG solutions, a homogeneous relaxation spectrum was observed, determined based on the master curve in the temperature range 0–60 °C. In this range, the viscoelastic properties could be scaled. A single maximum was observed in the relaxation spectrum. However, it was impossible to fully scale the viscoelastic properties of a 1% XG solution with temperature; the stress relaxation spectrum exhibited several local maxima, indicating a complex solution structure. Similarly, properties in the flow region [52], as represented by the time constant distributions of the De Kee model, indicated that XG solutions have more complex rheological properties than GG solutions. The hysteresis of the rheological properties (i.e., flow curves) of XG solutions was also observed under semi-industrial conditions (i.e., in situ experiments [53]).

## Figures and Tables

**Table 1:** The values of the power law model Equation (7) and the goodness of its fit as a value of $\chi^{2}$ for the semidilute solution of guar gum (0.5 g/dL), and the diluted (0.01 g/dL) and semidilute (0.05 g/dL) solutions of xanthan gum.

	GG	a	$R_{h}$, nm	$\chi^{2}$	XG	a	$R_{h}$, nm	$\chi^{2}$	XG	a	$R_{h}$, nm	$\chi^{2}$
20 °C	0.5	0.90	>1000	7.59 × $10^{-2}$	0.01	0.80	>1000	2.86 × $10^{-1}$	0.05	0.88	>1000	3.21$\times 10^{-2}$
30 °C		1.00	569	8.15 × $10^{-2}$		0.96	752	4.16 × $10^{-2}$		0.97	>1000	3.26 × $10^{-2}$
40 °C		0.98	342	6.65 × $10^{-2}$		0.89	334	5.74 × $10^{-2}$		0.68	>1000	8.66 × $10^{-2}$
50 °C		0.99	152	7.42 × $10^{-2}$		0.98	130	4.20 × $10^{-2}$		0.86	>1000	2.68 × $10^{-2}$
60 °C		1.00	68	2.60 × $10^{-2}$		0.97	84	4.36 × $10^{-2}$		0.96	629	2.78 × $10^{-1}$
70 °C		0.99	28	2.74 × $10^{-2}$		0.90	49	4.85 × $10^{-2}$		0.96	175	2.38 × $10^{-2}$
80 °C		1.00	16	2.84 × $10^{-2}$		0.99	34	3.73 × $10^{-2}$		1.00	68	2.41 × $10^{-2}$
90 °C		1.00	11	2.72 × $10^{-2}$		0.98	21	4.58 × $10^{-2}$		0.99	42	1.81 × $10^{-2}$

## Data Availability

The original contributions presented in this study are included in the article. Further inquiries can be directed to the corresponding author.
